# Rice Glycosyltransferase Gene *UGT85E1* Is Involved in Drought Stress Tolerance Through Enhancing Abscisic Acid Response

**DOI:** 10.3389/fpls.2021.790195

**Published:** 2021-12-23

**Authors:** Qian Liu, Guang-rui Dong, Yu-qing Ma, Shu-man Zhao, Xi Liu, Xing-kun Li, Yan-jie Li, Bing-kai Hou

**Affiliations:** The Key Laboratory of Plant Development and Environment Adaptation Biology, Ministry of Education, School of Life Science, Shandong University, Qingdao, China

**Keywords:** *UGT85E1*, glycosyltransferase, abscisic acid (ABA), reactive oxygen species (ROS), drought stress, rice

## Abstract

Drought is one of the most important environmental constraints affecting plant growth and development and ultimately leads to yield loss. Uridine diphosphate (UDP)-dependent glycosyltransferases (UGTs) are believed to play key roles in coping with environmental stresses. In rice, it is estimated that there are more than 200 *UGT* genes. However, most of them have not been identified as their physiological significance. In this study, we reported the characterization of a putative glycosyltransferase gene *UGT85E1* in rice. *UGT85E1* gene is significantly upregulated by drought stress and abscisic acid (ABA) treatment. The overexpression of *UGT85E1* led to an enhanced tolerance in transgenic rice plants to drought stress, while the *ugt85e1* mutants of rice showed a more sensitive phenotype to drought stress. Further studies indicated that *UGT85E1* overexpression induced ABA accumulation, stomatal closure, enhanced reactive oxygen species (ROS) scavenging capacity, increased proline and sugar contents, and upregulated expression of stress-related genes under drought stress conditions. Moreover, when *UGT85E1* was ectopically overexpressed in Arabidopsis, the transgenic plants showed increased tolerance to drought as well as in rice. Our findings suggest that *UGT85E1* plays an important role in mediating plant response to drought and oxidative stresses. This work may provide a promising candidate gene for cultivating drought-tolerant crops both in dicots and monocots.

## Introduction

Drought is one of the most important environmental cues that affect crop production. It is reported that drought stress will lead to a 50% yield reduction in the reproductive stage of the crops (Hu and Xiong, 2014; Lesk et al., 2016). Therefore, improving the drought tolerance of crops is an urgent need when humans face more and more serious water shortages.

Abscisic acid (ABA) has long been considered the most important plant hormone involved in drought stress responses (Schachtman and Goodger, 2008). When plant roots sense a lack of soil water, they produce large amounts of ABA. The increase of ABA in plant cells activates intracellular signal conversion and protein phosphorylation and then regulates the specific binding process of transcription factors such as ABF (ABRE-binding factor) and AREB (ABA-responsive element binding protein), inducing the expression of downstream stress response genes, which is the main mechanisms of drought resistance in plants (Lee and Luan, 2012; Daszkowska-Golec and Szarejko, 2013; Ali et al., 2018), namely the so-called ABA-dependent pathway. In addition, plants can also produce stress resistance through promoting root elongation, leaf curling, and helping plants maintain tissue water, namely the so-called ABA independent pathway (Hu and Xiong, 2014).

Under stress conditions, secondary metabolic regulation in plants is also an important way to cope with adverse environments, because the secondary metabolism of plants is the result of the plant interaction with the environments during plant evolution (Cook et al., 2004). For example, flavonoids, and anthocyanins increase in Arabidopsis when facing drought stress (Nakabayashi et al., 2014). UDP-Glycosyltransferases (UGTs) are a kind of enzyme that can catalyze the covalent addition of sugars to a broad range of secondary products and the UGT superfamily is believed to play crucial roles in modulating secondary metabolic balance in plant cells (Lim and Bowles, 2004; Bowles et al., 2005). Therefore, it would be very important to explore the role of UGTs in regulating metabolism-related stress responses. A previous study reported that *UGT79B2* and *UGT79B3* can glycosylate anthocyanins, and the overexpression lines accumulate more anthocyanins to adapt to drought stress (Li et al., 2017). Similarly, maize glycosyltransferase *UFGT2* modifies flavonols and contributes to plant acclimation to abiotic stresses (Li et al., 2018). A rice glycosyltransferase *UGT83A1* glycosylates most of the lignin precursors and flavonoids, its overexpressing lines showed strong resistance to salt, drought, and cold stress (Dong et al., 2020). As important signaling molecules, phytohormones are also regulated by molecular glycosylation to address the stress responses (Bowles et al., 2006; Wong et al., 2006). For example, *UGT71C5* and *UGT75B1* are UGT genes that target ABA in Arabidopsis. Their knock-out mutants in the seedling stage showed higher resistance than the wild type to salt stress (Liu et al., 2015; Chen et al., 2020). Glycosylation of auxin also affects plant stress responses. *UGT74E2*-mediated glycosylation of IBA enhances drought stress resistance in Arabidopsis (Tognetti et al., 2010). Secondary metabolism-linked UGTs are indispensable in regulating plant stress responses, although most current studies in this aspect are centered on the model plant Arabidopsis.

In rice, it is estimated that there would be more than 200 UGT genes. However, most of them have not been identified as their physiological significance. In this study, we reported the characterization of a rice glycosyltransferase gene, *UGT85E1*. The *UGT85E1* gene is significantly upregulated by drought stress and ABA treatments. The overexpression of *UGT85E1* led to the enhanced tolerance in transgenic rice plants to drought stress, while the rice *ugt85e1* mutants showed the more sensitive phenotype to drought stress. *UGT85E1* overexpression induced stomatal closure, ABA accumulation, and enhanced reactive oxygen species (ROS) scavenging capacity under drought stress conditions. When *UGT85E1* was ectopically overexpressed in Arabidopsis, the transgenic lines showed increased tolerance to drought conditions as well as in rice. Our findings suggest that glycosyltransferase gene *UGT85E1* plays an important role in mediating plant response to drought and oxidative stresses. This work may provide a promising candidate gene for cultivating drought-tolerant crops both in dicots and monocots.

## Results

### *UGT85E1* Gene Is Induced by Mannitol and ABA

**Figure 1:**
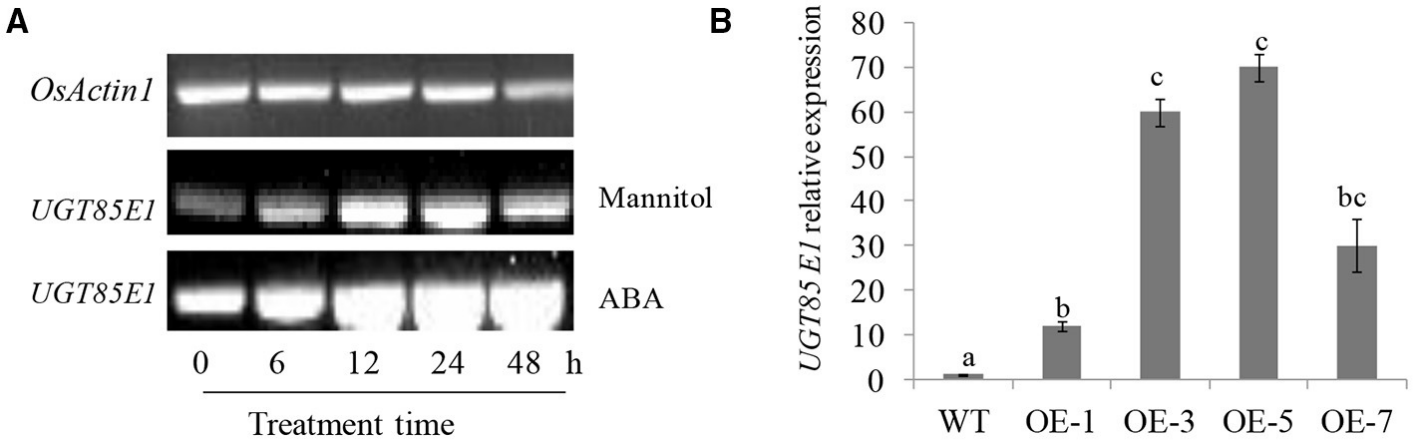
The induced expression of *UGT85E1* and the preparation of *UGT85E1* transgenic plants. **(A)** *UGT85E1* transcript level was up-regulated by 200 mM Mannitol and 100 μM ABA treatments through reverse transcription-PCR (RT-PCR) analysis; **(B)** Generation and identification of *UGT85E1* overexpression lines in rice. *OsActin1* was used as the internal control. Error bars indicate the SD of three independent experiments. Statistically significant differences were determined by one-way ANOVA, followed by Tukey's test (*P* < 0.05).

In our attempt to screen rice UGT genes responsive to abiotic stress, *UGT85E1* (LOC_Os02g51900) drew our attention because of its response to drought stress. We examined the responsiveness of *UGT85E1* to mannitol and exogenous ABA *via* real-time quantitative reverse transcription PCR (qRT-PCR). The expression level of *UGT85E1* under 200 mM mannitol treatment gradually increased from 6 to 24 h, decreasing slightly at 48 h. The induction of *UGT85E1* was also observed from 6 to 48 h under 100 μM ABA treatment (Figure 1A). These two treatments significantly induced the expression of *UGT85E1*, suggesting that this gene may be involved in drought stress. To analyze the function of *UGT85E1*, we created the overexpression lines and mutant lines of the *UGT85E1* gene. Two overexpression lines (OE-3 and OE-5) and two CRISPR/cas9 mutant lines (ko-8 and ko-9) were subjected to drought stress for further analysis (Figure 1B, Supplementary Figure 1).

### The Phenotype of *UGT85E1* Transgenic Plants Under Drought Stress

**Figure 2:**
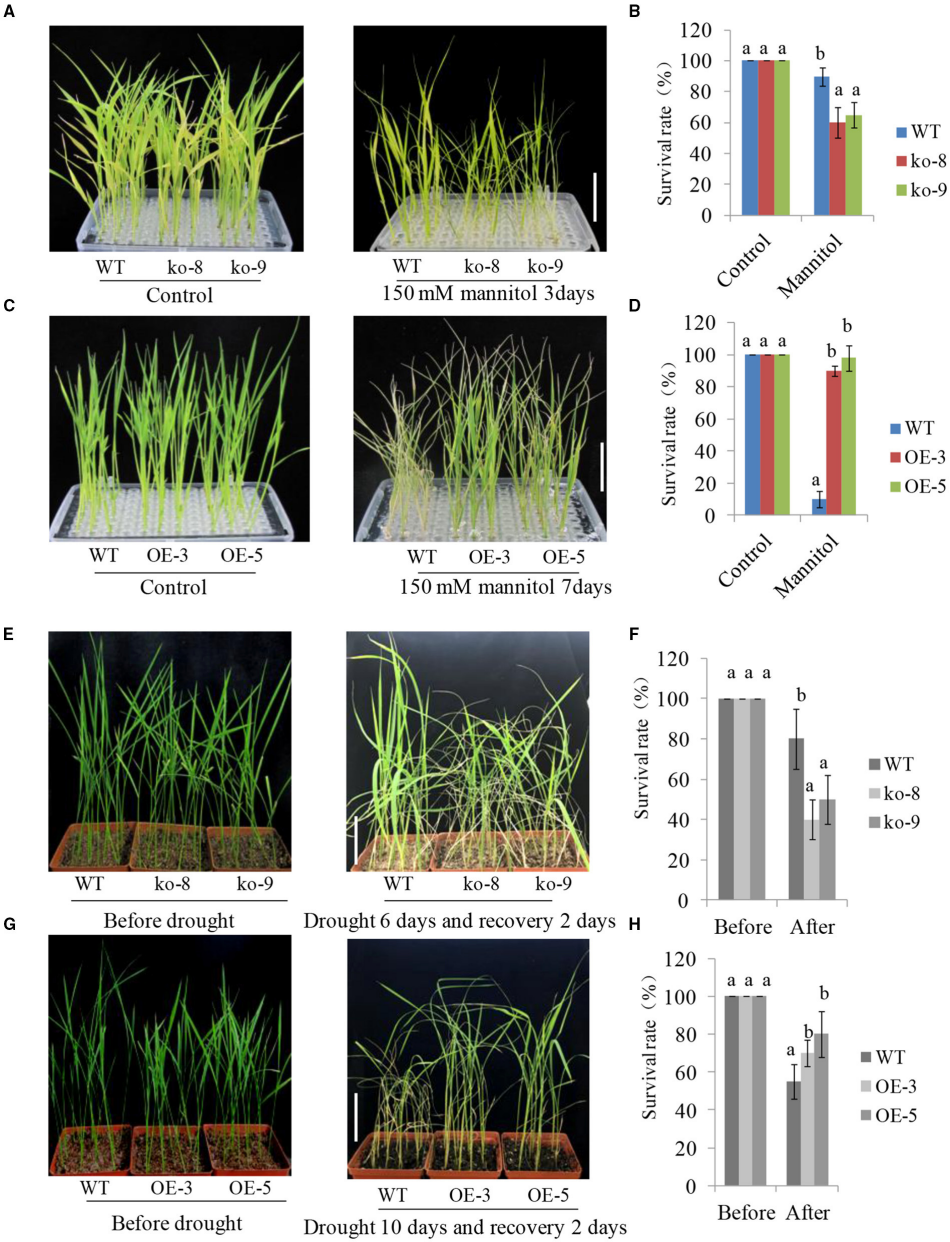
Growth phenotypes of *UGT85E1* overexpression lines and mutants under mannitol and drought treatments. **(A,C)** Phenotypes of *UGT85E1* mutants and overexpression lines after 150 mM Mannitol treatment, scale bar = 5 cm; **(B,D)** The survival rate of mutant lines and overexpression lines after 150 mM Mannitol treatment; **(E,G)** Phenotypes of *UGT85E1* mutants and overexpression lines were grown in the soil after drought treatment for 6–10 days and recovery for 2 days, scale bar = 8 cm; **(F,H)** The survival rate of mutants and overexpression lines after drought treatment. Error bars indicate the SD of three independent experiments. Statistically significant differences were determined by one-way ANOVA, followed by Tukey's test (*P* < 0.05).

Under normal conditions, 2-week-old *UGT85E1* overexpression lines and mutants showed normal and similar growth phenotypes with wild-type plants (WT). After 150 mM mannitol treatment, most mutants appeared more withered than the WT, however, the two overexpression lines performed better growth than WT (Figures 2A,C). Survival rates showed consistency with the phenotype observation (Figures 2B,D). We also conducted drought treatments using 4-week-old mutants and overexpression lines. No growth difference of mutants and overexpression lines when compared with WT can be observed under soil culture and normal irrigation conditions. After deprival of irrigation for about 1.5 weeks, some mutant plants wilted. After re-irrigating for 2 days, the mutants were more withered and showed a lower survival rate than that of WT (Figures 2E,F). However, the overexpression lines grew better than that of WT under drought conditions, showing more green, upright petiole, and a much higher survival rate (Figures 2G,H). These experiments proved that *UGT85E1* is involved in drought stress response and its overexpression can significantly enhance the drought tolerance of rice.

### *UGT85E1* Is Involved in the Modulation of Endogenous ABA Level and Stomatal Aperture

**Figure 3:**
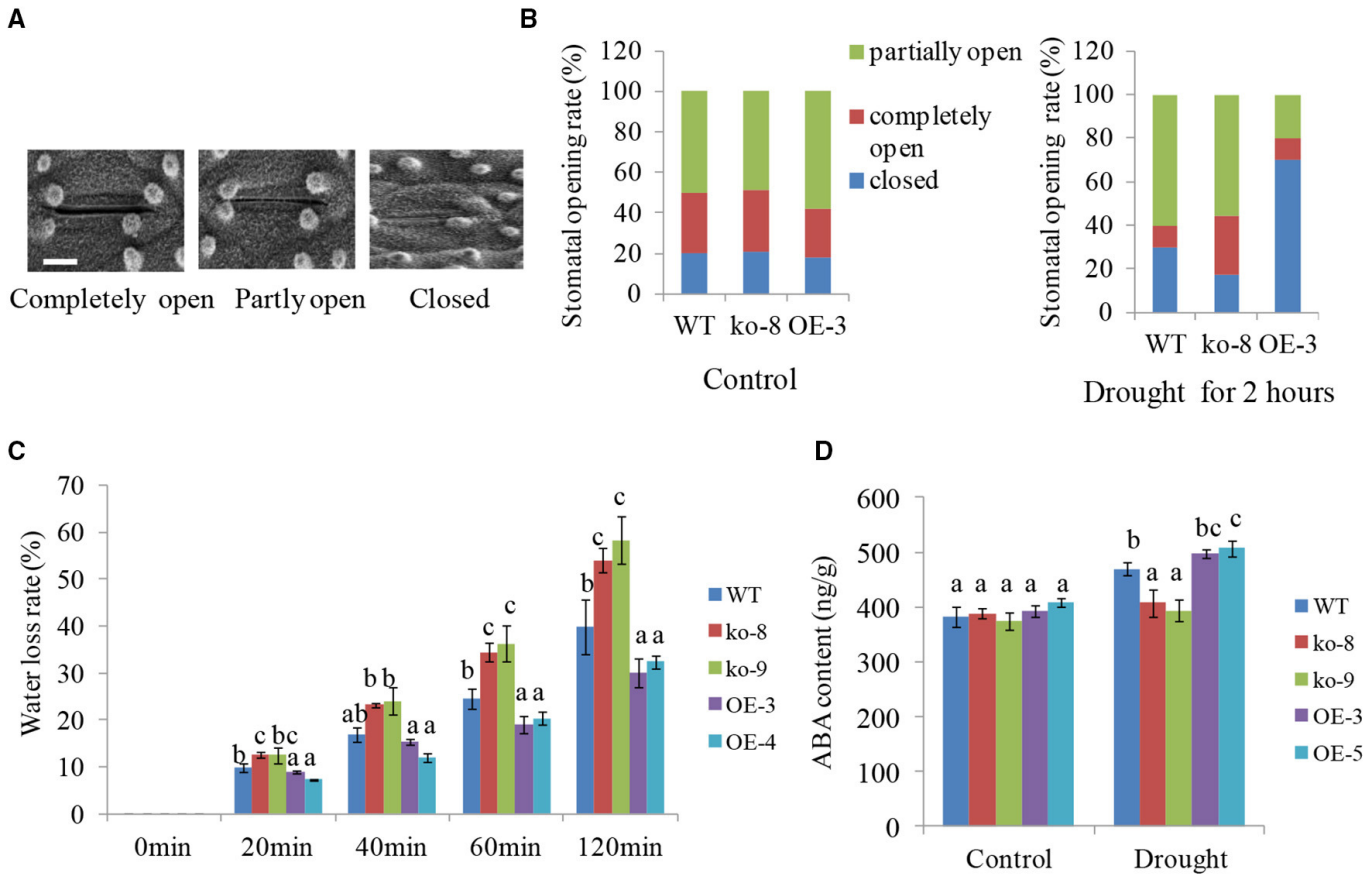
Stomatal aperture, Water loss rates, and abscisic acid (ABA) levels in *UGT85E1* transgenic plants under drought treatment. **(A)** A scanning electron microscope was used to observe stomatal opening. Bar = 5μm; **(B)** The ratio of stomatal opening and closing of WT and *UGT85E1* transgenic lines before and after drought. **(C)** Water loss rates of detached *UGT85E1* transgenic leaves within 120 min. **(D)** Detection of ABA content in WT and *UGT85E1* transgenic lines under normal and drought stress conditions. Error bars indicate the SD of three independent experiments. Statistically significant differences were determined by one-way ANOVA, followed by Tukey's test (*P* < 0.05).

Upon exposure to drought stress, water loss of plant leaves mainly occurred through the stomatal opening. Thus, we investigated the stomatal aperture of 4-week-old different transgenic lines under dehydration conditions. Here, the stomatal opening was classified into three types, i.e., fully open, partially open, and completely closed as shown in Figure 3A. Before dehydration, the ratio of three types of stomatal opening is roughly the same between WT, mutants, and overexpression lines. After 2 h of dehydration of detached leaves, however, the proportion of stomatal closure increased and the proportion of partially open stomata significantly decreased in overexpression line OE-3 compared with WT (Figure 3B). The mutant line ko-8 was on the contrary with less closed and more open stomata compared to WT. For example, the percentage of completely closed stomata in WT, ko-8, and OE-3 lines was 30.1, 17.3, and 70%, respectively (Figure 3B). This result showed that the drought tolerance of *UGT85E1* transgenic rice was due to the reduced stomatal opening.

Then, we detected the rate of water loss from detached leaves of each *UGT85E1* transgenic line within 120 min. The result indicated that the two overexpression lines exhibited lower water loss than that of WT, but the mutants showed more water loss (Figure 3C).

**Figure 4:**
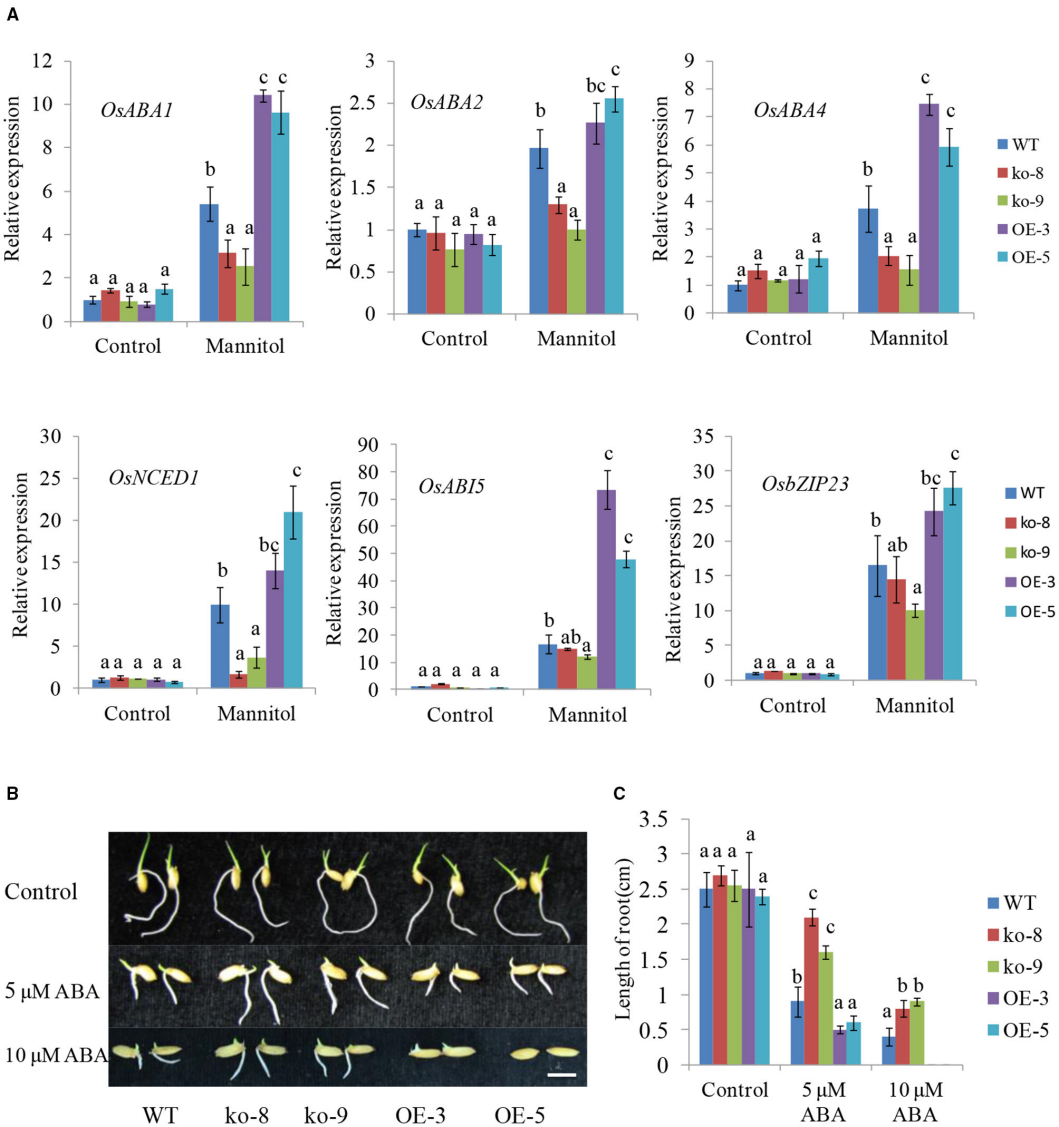
Expressions of ABA-related genes and ABA responses in *UGT85E1* transgenic plants under normal and treatment conditions. **(A)** Real-time PCR analysis of the expression of ABA biosynthesis and responsive genes under normal and mannitol treatment conditions; **(B)** Influence of ABA treatments on post-germination growth of UGT85E1 transgenic rice seedlings; **(C)** Rice root lengths of *UGT85E1* transgenic lines were recorded under ABA treatments and control condition. Bars = 0.5 cm. Error bars indicate the SD of three independent experiments. Statistically significant differences were determined by one-way ANOVA, followed by Tukey's test (*P* < 0.05).

It is well-known that ABA can induce stomatal closure. We thus examined the endogenous ABA levels in *UGT85E1* transgenic lines and WT under normal and drought stress conditions. Although ABA levels were similar for different genotypic plants under control conditions, overexpression lines showed much higher ABA levels than WT under drought stress, whereas the mutants showed lower ABA levels (Figure 3D). This result prompted us to hypothesize that *UGT85E1* may be involved in regulating ABA biosynthesis in drought stress. To confirm this, the transcript levels of ABA biosynthesis and responsive genes were analyzed. As shown in Figure 4A, the expression levels of ABA biosynthesis genes *OsABA1, OsABA2, OsABA4*, and *OsNCED1* and ABA-responsive genes *OsABI5* and *OsbZIP23* were highly upregulated by drought stress in the *UGT85E1* overexpression lines as compared with WT, while in the mutants, they were down-regulated. The upregulation or downregulation of ABA biosynthesis and responsive genes would change the sensitivity of seed germination to exogenously applied ABA. As shown in Figures 4B,C, our investigation indicated that the root length of overexpression lines was shorter than WT seedling under 5–10 μM of ABA treatment, whereas the mutants showed the longer root. These results suggest that the role of *UGT85E1* in enhancing drought tolerance may be due to the enhanced ABA response in rice.

### Overexpression of *UGT85E1* Enhances ROS Scavenging in Transgenic Plants

**Figure 5:**
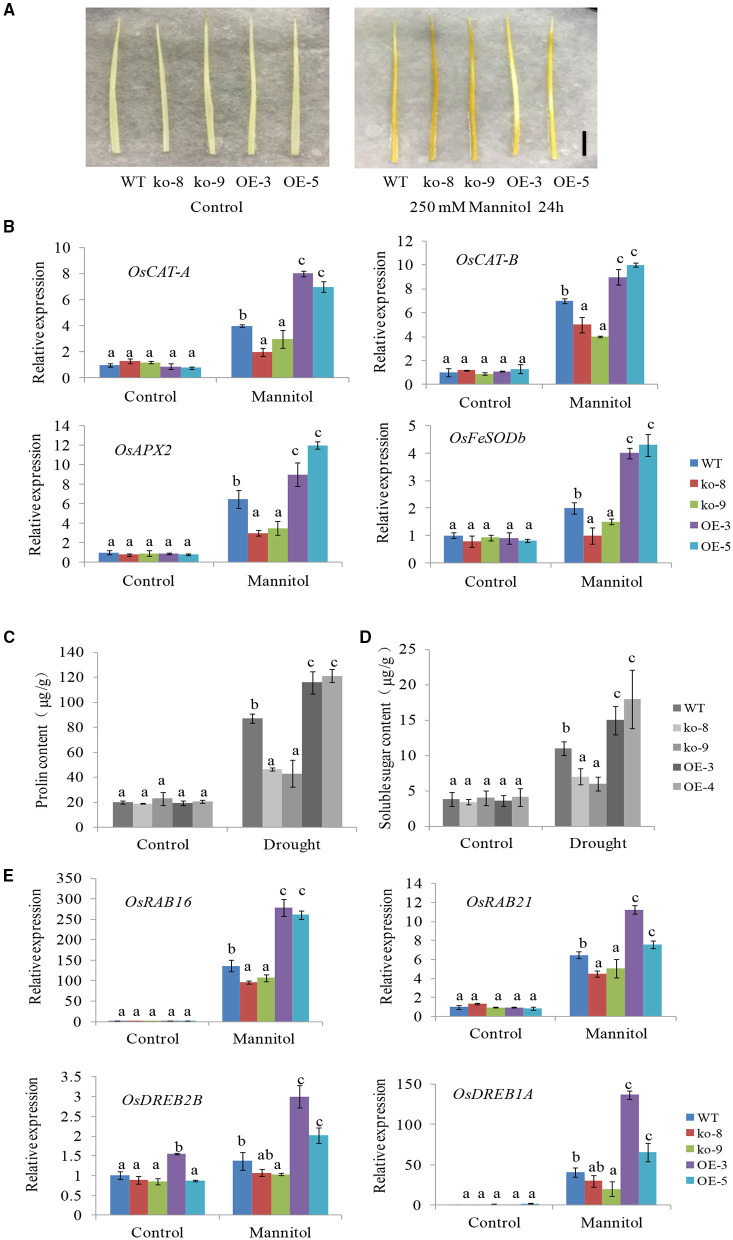
Accumulation of hydrogen peroxide (H_2_O_2)_ and the expression levels of reactive oxygen species (ROS) scavenging genes and drought stress response genes in *UGT85E1* transgenic lines. **(A)** DAB staining of *UGT85E1* transgenic lines under control and mannitol treatments, scale bar = 2 cm; **(B)** Transcription levels of ROS scavenging enzyme genes in *UGT85E1* transgenic lines under normal and drought stress conditions; **(C,D)** Proline and soluble sugar contents in rice leaf tissues sampled from *UGT85E1* transgenic lines and WT plants under control and drought treatment conditions. **(E)** Expression levels of drought stress response genes in *UGT85E1* transgenic lines. Error bars indicate the SD of three independent experiments. Statistically significant differences were determined by one-way ANOVA, followed by Tukey's test (*P* < 0.05).

Stresses often lead to the accumulation of hydrogen peroxide (H_2_O_2_) in plant cells, and the ability of cells to remove H_2_O_2_ reflects the level of resistance to stresses (Li et al., 2004). It was reported that ABA could significantly promote the activities of ROS scavenging enzymes such as SOD (Superoxide dismutase), APX (Ascorbate peroxidase), and CAT (Catalase) under stress conditions (Jiang and Zhang, 2002; Xu et al., 2018). Given that *UGT85E1* is involved in ABA response as mentioned above, we thus examined ROS accumulation and the relative expression of several genes encoding ROS scavenging enzymes in *UGT85E1* overexpression lines and mutants. 3,3′-Diaminobenzidine (DAB) staining indicated that H_2_O_2_ accumulated more in *ugt85e1* mutants than WT, the overexpression lines showed the opposite changes under mannitol treatment (Figure 5A). Transcript levels of several genes encoding ROS scavenging enzymes were significantly upregulated in overexpression lines, but down-regulated in *ugt85e1* mutant lines compared with WT after 250 mM mannitol treatment (Figure 5B). These results indicated that *UGT85E1* overexpression promotes the scavenging of ROS.

As osmoprotective molecules, proline and sugar are recognized to protect cells against osmotic stress. Numerous studies have demonstrated that the proline and sugar accumulation is enhanced in response to different abiotic stresses and also by the induction of ABA (Verslues et al., 2007; Szabados and Savoure, 2010; Vishwakarma et al., 2017; Živanović et al., 2020). Here, we measured the contents of proline and soluble sugar in the *UGT85E1* overexpression lines and mutants subjected to drought treatment. Our results showed that much more proline and soluble sugar were accumulated in OE-3 and OE-5 plants, but less in ko-8 and ko-9 compared with WT (Figures 5C,D).

We also investigated the expression changes of four stress-related genes (*OsRAB16, OsRAB21, OsDREB2B*, and *OsDREB1A*) after 250 mM mannitol treatment. The results showed that the expression levels of four genes in overexpression lines were significantly higher than that in WT, their expression levels in mutant lines were lower than that in WT (Figure 5E). The above-mentioned results suggest that the *UGT85E1* gene exerts its important influence on these stress-related genes, directly or indirectly, and plays a broad role when facing stress conditions.

### *UGT85E1* Can Enhance Oxidative Stress Tolerance in Detached Rice Leaves

**Figure 6:**
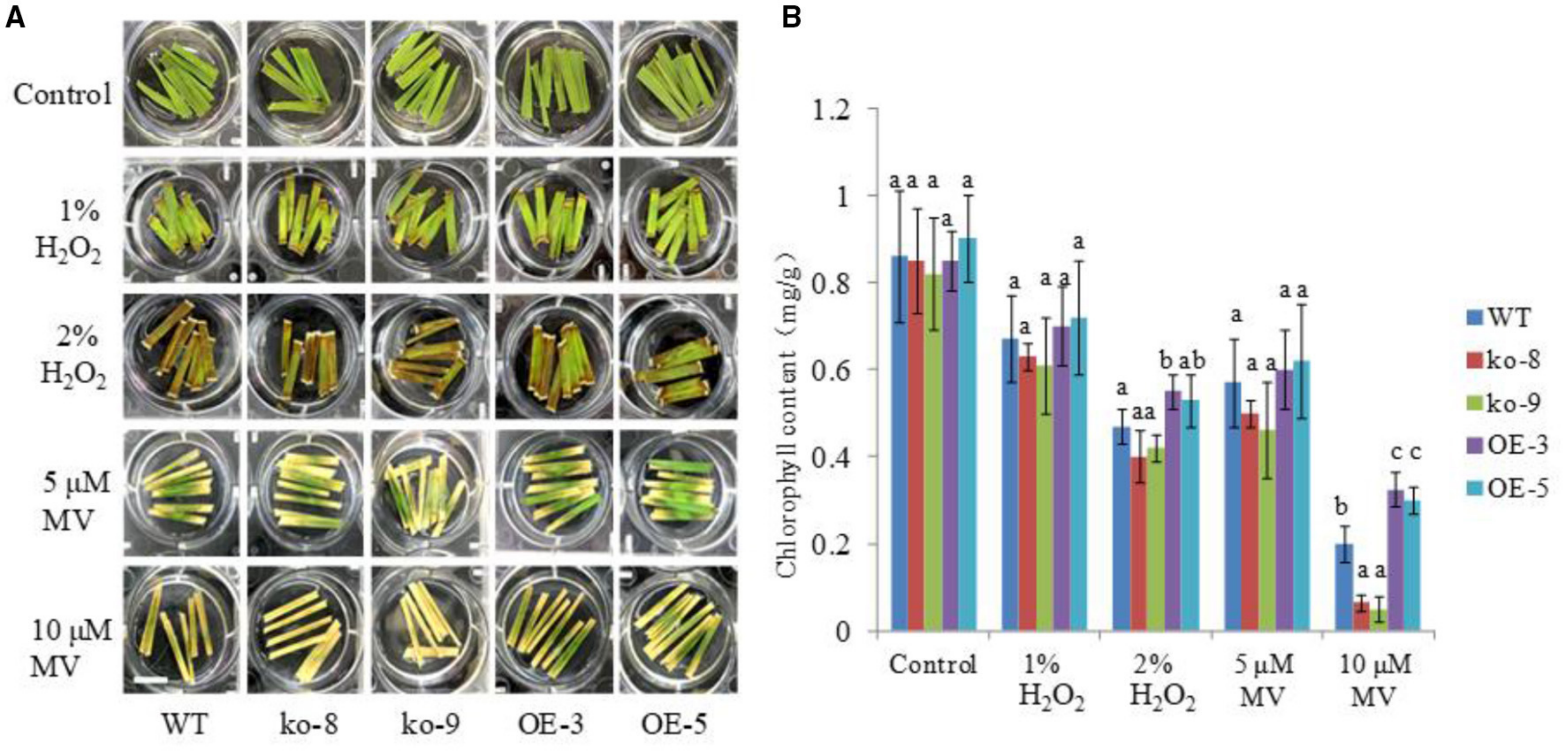
Effects of H_2_O_2_ and methyl viologen (MV) treatments on the detached leaves of *UGT85E1* transgenic lines. **(A)** Effects of different concentrations of H_2_O_2_ and MV on the leaves of *UGT85E1* transgenic lines, bar = 2 cm; **(B)** Determination of the chlorophyll contents of WT and *UGT85E1* transgenic lines after H_2_O_2_ and MV treatments. Error bars indicate the SD of three independent experiments. Statistically significant differences were determined by one-way ANOVA, followed by Tukey's test (*P* < 0.05).

To further investigate the role of *UGT85E1* in scavenging ROS, we also tested the detached leaves of the overexpression lines and mutant lines with the exogenously applied H_2_O_2_ and herbicide methyl viologen (MV), which can generate ROS locally in chloroplasts. As shown in Figure 6A, under the control condition, there was no difference in the green color of the detached leaves from each of the lines soaked in distilled water for 1 week. When treated with 2% H_2_O_2_ or 10 μM MV, we can see that most overexpression samples were greener than WT, and mutant samples were browner than WT. The determination of chlorophyll contents also confirmed this observation (Figure 6B). This result again demonstrated that the overexpression of *UGT85E1* can enhance the ability to scavenge H_2_O_2_ and to resist oxidative stress.

### Ectopic Expression *UGT85E1* in Arabidopsis Significantly Enhances Drought Stress Tolerance

**Figure 7:**
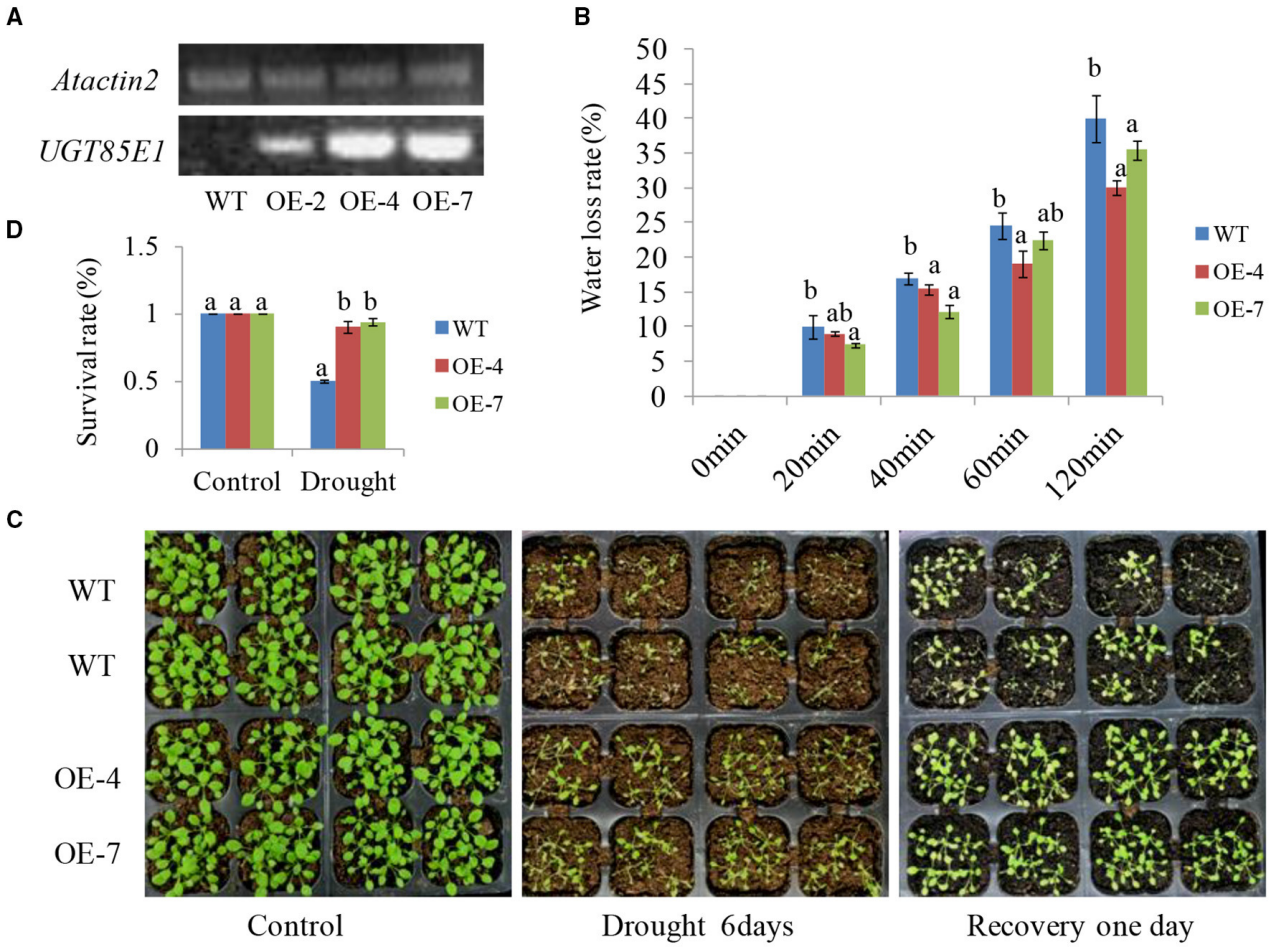
Drought-tolerant phenotype of *UGT85E1* transgenic Arabidopsis. **(A)** Identification of overexpressing *UGT85E1* transgenic Arabidopsis lines through RT-PCR, the *Atactin2* was used as the internal control; **(B)** Detection of water loss rates of detached leaves from *UGT85E1* transgenic Arabidopsis lines within 120 min; **(C)** Growth phenotype *UGT85E1* transgenic lines after drought treatment for 6 days and recovery for 1 day with water; Untreated plants were used as the control; **(D)** Survival rates of *UGT85E1* transgenic Arabidopsis lines and wild-type WT plants after drought treatment were examined. Error bars indicate the SD of three independent experiments. Statistically significant differences were determined by one-way ANOVA, followed by Tukey's test (*P* < 0.05).

To further verify the effect of *UGT85E1* in enhancing drought stress tolerance, we overexpressed *UGT85E1* in Arabidopsis and investigated the drought tolerance using two overexpression lines OE-4 and OE-7 (Figure 7A). Dehydration assay indicated that Arabidopsis OE-4 and OE-7 had a lower water loss rate than WT within 120 min (Figure 7B). When 3-week-old soil-grown seedlings were subjected to drought stress for 1 week and followed by re-irrigation for 24 h, we can see that the overexpression lines grew better than WT. Not only the leaves of OE-4 and OE-7 were fully extended but almost all survive. However, about half of the WT wilted (Figures 7B,C,D). These results suggest that *UGT85E1* overexpression in Arabidopsis also contributes to drought tolerance.

**Figure 8:**
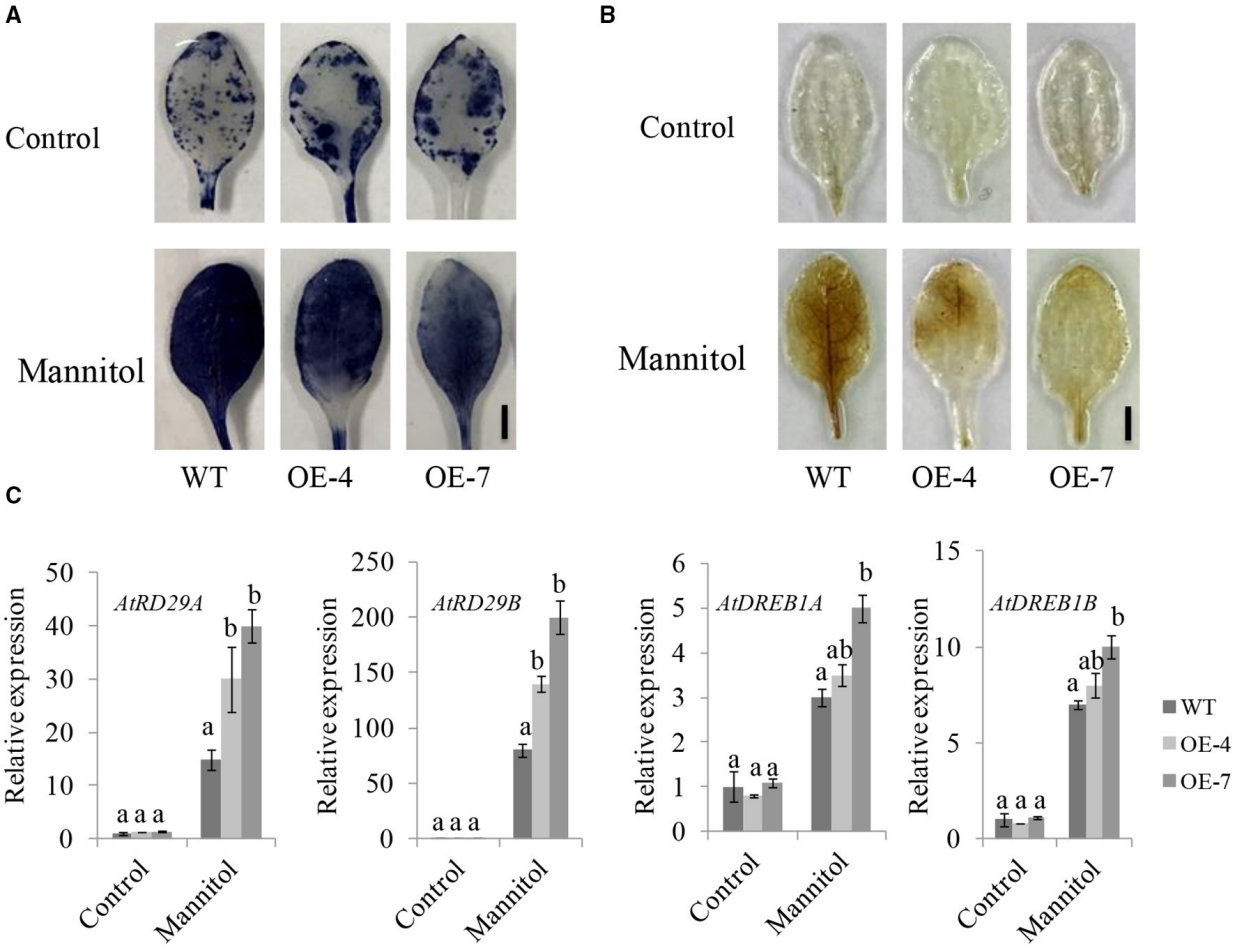
Overexpression of *UGT85E1* enhanced antioxidant ability of Arabidopsis plants. **(A,B)** Antioxidant capacity *UGT85E1* transgenic lines assessed by nitroblue tetrazolium test (NBT) **(A)** and 3,3′-Diaminobenzidine (DAB) **(B)** staining after 250 mM mannitol treatment for 12 h, untreated plants were used as the control. **(C)** Expression levels of stress response genes were analyzed by Real-time qRT-PCR for *UGT85E1* transgenic Arabidopsis lines under Mannitol treatment. Error bars indicate the SD of three independent experiments. Statistically significant differences were determined by one-way ANOVA, followed by Tukey's test (*P* < 0.05).

We also investigated the ROS levels in Arabidopsis overexpression lines and WT plants under stress conditions. The DAB and nitroblue tetrazolium test (NBT) staining indicated that ROS accumulated much less in *UGT85E1* transgenic Arabidopsis lines than in WT (Figures 8A,B). We further analyzed the expression of the abiotic stress-related genes and found that the transcript levels of At*RD29A*, At*RD29B*, At*DREB1A*, and At*DREB1B* in transgenic Arabidopsis lines were higher than in WT (Figure 8C). These ectopic expression data provide more powerful evidence for the involvement of *UGT85E1* in enhancing drought stress tolerance.

## Discussion

Rice is the most important food in the world that feeds more than half of the world's population. However, with the deterioration of the current global environment and uneven precipitation, the area of soil drying increased gradually. To meet the demand for food, cultivating rice varieties with drought tolerance would be very important. In this study, we characterized the *UGT85E1* gene, a putative UGT gene that is involved in drought response. The transcript level of *UGT85E1* was strongly induced by mannitol and ABA treatments. Its overexpression lines showed an enhanced survival rate and improved growth performance under mannitol treatment or drought treatment conditions. When it was introduced into Arabidopsis, the ectopically expressed *UGT85E1* substantially enhanced drought tolerance. These findings suggest the *UGT85E1* has promising application in the genetic improvement of crops with abiotic stress resistance.

Abscisic acid (ABA) is a key regulator of both plant development and stress response, including seed germination and dormancy, root development, stomatal movement, and abiotic stress tolerance (Jeon et al., 2010; Watkins et al., 2017). Following these above findings, *UGT85E1* overexpression plants had the increased ABA level with enhanced drought tolerance, more closed stomata, and less water loss rate than that in WT plants when confronting drought stress. ROS at the physiological level is believed to act as a signaling molecule that regulates plant adaptation to various stresses, while ROS at an excessive level often causes significant damage to plant cells, leading to deleterious effects on stress tolerance (Choudhury et al., 2013; Xie et al., 2014; You and Chan, 2015; Tognetti et al., 2017). ABA plays a crucial role in ROS homeostasis and it can regulate the expression of ROS producing and scavenging genes. For example, abiotic stress-induced ABA accumulation triggers the increased generation of ROS and upregulates the activities of antioxidant enzymes in maize leaves (Jiang and Zhang, 2002). Arabidopsis glutathione peroxidase genes are also regulated by abiotic stresses together with ABA (Milla et al., 2003). Antioxidant enzymes were often involved in the stress responses of plants. For instance, the ascorbate peroxidase gene *OsAPX2* plays a positive role in chilling tolerance by enhancing H_2_O_2_ scavenging (Zhang et al., 2013). *OsCATB* prevents the excessive accumulation of H_2_O_2_ under water stress (Ye et al., 2011). Our results showed that the expression levels of several ROS scavenging-related genes clearly increased in the *UGT85E1* overexpression plants and decreased in mutant lines. Consistent with this, the *UGT85E1* overexpression lines exhibited better growth under oxidative stress caused by the direct application of H_2_O_2_ or MV treatment. In contrast, mutant plants showed enhanced sensitivity to oxidative stress. These results suggest that the improved drought tolerance of *UGT85E1* overexpression plants may be due to the ABA-dependent ROS scavenging. In addition, more proline and soluble sugar as osmotic protective compounds were also accumulated in *UGT85E1* overexpression plants. Previous reports demonstrated that salinity-induced proline accumulation is dependent on ABA, which is also consistent with the proposal that proline is associated with redox regulation and might serve as an antioxidant (Van Breusegem and Dat, 2006; Hoque et al., 2008). All of these observations highlight a link between the altered ABA responses and the changed H_2_O_2_ levels in *UGT85E1* transgenic plants.

Moreover, *UGT85E1* gene expression also promotes the upregulation of multiple stress-related genes in rice and Arabidopsis under drought conditions, including ABA biosynthesis and signaling genes. But the molecular mechanism of *UGT85E1* involved in drought tolerance is not known. Because UGT85E1 is a putative UDP-dependent glycosyltransferase, which mainly catalyzes the glycosylation of plant secondary products, its influence on the expression levels of ABA biosynthesis and signaling genes would depend on its biochemical function. Unfortunately, the relevant substrate(s) has not been identified yet in this study, although we examined a lot of secondary plant metabolites. Thus, the precise mechanism for how UGT85E1 functions is not known at the moment. Here, we can suppose several possible action modes of UGT85E1. The first possibility is that UGT85E1 glycosylates ABA-antagonistic factors such as cytokinins. Several studies have revealed the interplay between cytokinins and ABA, which act antagonistically in regulating stress responses. For instance, the *trans*-zeatin riboside decreases significantly in the sunflower plants, while the endogenous ABA content was increased when suffering from drought stress (Hansen and Dörffling, 2003). AHK2, AHK3, and A-type ARRs act as negative regulators in the cold stress signaling pathways *via* inhibiting ABA response (Jeon et al., 2010). It is also reported that cytokinin-deficient *ipt1357* mutant and *CKX* overexpression lines are more sensitive to ABA compared with the wild type, leading to a higher induction of ABA signaling genes by stresses and the enhanced stress tolerance (Nishiyama et al., 2011). A comparative genome-wide analysis of the leaves of *arr1,10,12* and WT plants under dehydration conditions suggested a cytokinin signaling-mediated network controlling plant adaptation to drought *via* many ABA-responsive genes (Nguyen et al., 2016). If UGT85E1 glycosylates some unidentified cytokinin(s), the removal of the antagonistic effect would promote the expression levels of ABA biosynthetic and signaling genes, leading to enhanced drought tolerance. The second possibility is that UGT85E1 glycosylates some inhibitor(s) of some transcription factors and relieves the inhibition effect on the transcription of ABA biosynthetic and signaling genes. In addition, we can also not exclude the possibility that UGT85E1 may glycosylate the metabolites of ABA and regulate ABA synthesis by a feedback mechanism, just like the case of SA synthesis regulation by UGT76D1 (Huang et al., 2018). It is estimated that there are more than 200 UGTs in rice (Caputi et al., 2012). UGT85E1 is close to the UGT85 family in Arabidopsis. It is reported that UGT85A1 is responsible for glycosylating *trans*-zeatin which is a kind of cytokinin (Hou et al., 2004; Jin et al., 2013). If UGT85E1 can also glycosylate cytokinins, it would result in decreased cytokinins and increased ABA responses in overexpression lines, which is consistent with our observation in this study. However, no significant catalytic activity of UGT85E1 was found toward typical cytokinins such as *trans*-zeatin, *cis*-zeatin, and isopentenyl adenine in our biochemical assays (data not shown). We also detected some other secondary natural products related to abiotic stresses, for instance, anthocyanins, flavonoids, phenolic acids, phenylpropanoids, auxins, ABA, etc. But still, no catalytic activity was found under our experimental conditions. Clearly, it requires further investigation into this aspect. The identification of substrate(s) would provide useful cues into the complex mechanism of UGT85E1 in plant abiotic stress responses.

## Materials and Methods

### Plant Materials and Growth Conditions

The WT rice plant and also the genetic background of the transgenic rice plants were ZH11 (*Oryza sativa* L. subsp. *japonica* cv. Zhonghua No.11). Rice seedlings were hydroponically grown or soil-grown in a growth chamber under standard growth conditions. For *Arabidopsis thaliana*, the ecotype Columbia 0 (Col-0) was used as wild type and transformed by the constructed plasmids. Seeds were sown on Murashige and Skoog (MS) medium, cold-treated for 3 days at 4°C, and transferred to controlled growth chamber under 16 h light/ 8 h dark condition with a fluency rate of 100 μmol/s/m^2^ of white light (produced by cool-white fluorescent lamps) at 22°C.

### Vector Construction and Plant Transformation

To construct the rice overexpression vector, the full-length cDNA (1,461 bp) of *UGT85E1* were amplified and cloned into pUN1301 binary vector under the control of maize ubiquitin promoter. For the ectopic expression of *UGT85E1* in Arabidopsis, the full-length cDNA of *UGT85E1* were cloned into a pBI121 binary vector under the control of CaMV 35S promoter. For the knocking-out of *UGT85E1* in rice, the CRISPR/Cas9 BGK01 kit was purchased from Biogle Biotechnology Company (Hanghzhou, China) (http://www.biogle.cn) and the knocking-out vector was constructed according to the instructions. Rice transformation was carried out by the *Agrobacterium*-mediated method (Hiei et al., 1994), and the flower dipping method was used for Arabidopsis transformation (Clough and Bent, 1998).

### Stress Assays of the Transgenic Plants

For the hydroponic treatment, rice seedlings grown for 3 days were treated with 150 mM mannitol for 1 week. Non-treated seedlings were used as the control. For the drought stress treatment of soil-grown seedlings, uniformly growing seedlings were transplanted to the soil and grown for 4 weeks under normal irrigation conditions. Then, irrigation was stopped for about 10 days and re-irrigation was provided. After the recovery for 2 days, pictures of growth phenotypes were taken and the survival ratio (the number of surviving plants over the total number of treated plants in the pot) of each line was calculated. For oxidative stress tolerance assays, leaf disks from 3-week-old seedlings were soaked into H_2_O_2_ or MV according to the indicated concentrations for 5 days, those leaf discs soaked in water were used as control.

To test the ABA sensitivity at the germination stage, seeds of the *UGT85E1* transgenic lines were germinated on 6-well plates containing 5 μM or 10μM ABA, the deionized water was used as the control. These seeds were germinated under 10 h light/14 h dark condition at 28°C in a growth chamber for 5 days, and then the root length of each seedling was measured.

For the drought treatment of *UGT85E1* transgenic Arabidopsis, 3-week-old soil-grown seedlings were kept without irrigation for 6 days and then again provided with watering. After recovery for 24 h, the survival rate was calculated and growth performance was photographed.

### Measurement of Stomatal Closure in Response to Drought Treatment

The detached leaves of 3-week-old rice were placed in the air for 2 h. Water is naturally lost to promote stomatal closure. Firstly, the leaves were fixed with 2.5% glutaraldehyde fixator at 4°C for 12–16 h, and then washed with 1×phosphate buffered saline (PBS) for 5 times; Secondly, gradient ethanol dehydration, and then drying the samples in the dryer, spraying gold in ion sputtering device, finally a scanning electron microscope (FEI Quanta250 FEG, Oregon, USA) was used to observe and take photos.

### Determination of ABA Content

Leaves of 2-week-old *UGT85E1* transgenic rice and WT plants were detached and kept in the air for 2 h. Approximately 50 mg of leaf tissues were collected and rapidly frozen with liquid nitrogen, followed by homogenizing the sample in PBS (PH7.4). Samples were centrifuged for 20 min, and the supernatant was collected carefully for determination of ABA. The ABA concentration was assayed according to the instructions provided by the plant hormone abscisic acid ELISA Kit (Shanghai Fusheng Industrial Co., Ltd, Catalog number: A112641-96T, Shanghai, China).

### Physiological Measurements

For calculation of water loss rate, the detached leaves of the 3-week-old rice plants were firstly weighed to record the fresh weight (FW), and then the samples were dehydrated in the air. The samples were weighed every 20 min to record the dry weight (DW; Xiong et al., 2014). Ten plants of each line were used in each replicate with three replicates. The water loss rate is defined as the (FW-DW)/FW×100%. For the determination of H_2_O_2_ accumulation under drought stress, 3-week-old rice plants or 4-week-old Arabidopsis plants were treated with 250 mM mannitol for 24 h. The leaves were subjected to 0.1% DAB staining for 16 h (pH = 5.8), followed by incubation in the de-staining buffer (ethanol/lactic acid/glycerol=3:1:1; Thordal-Christensen et al., 1997). Similarly, NBT-staining for superoxide detection was conducted as described by Wohlgemuth et al. (2002).

To determine the content of proline and soluble sugar, the detached leaves of 4-week-old rice plants were dehydrated at room temperature for 1 h, the non-treated plants were served as control 0.1 g of leaves were homogenized with 2 ml 3% sulfosalicylic acid and followed by centrifugation. 1 ml supernatant was taken and an equal volume of glacial acetic acid and ninhydrin were added, followed by inoculation in boiling water bath for 60 min. After cooling, 3 ml toluene was added and the content of proline was determined at 520 nm (Nakazawa et al., 1982). Similarly, to measure the content of soluble sugar, 0.1 g of the harvested leaves were homogenized in 2 ml distilled water and boiled for 30 min, followed by centrifugation. About 1 ml supernatant was taken, and 2 ml anthrone and 2 ml sulfuric acid were added, followed by a full mix and inoculation in boiling water bath for 10 min, then soluble sugar was measured at 620 nm (Morris, 1948).

### RNA Extraction and qRT-PCR Analysis

For analyzing the induced expression of *UGT85E1*, 2-week-old hydroponically grown rice seedlings were subjected to treatments with 200 mM mannitol or 100 μM ABA, and the leaf tissues were harvested at 0, 6, 12, 24, and 48 h time points. For detecting expression levels of the ABA biosynthesis and signal genes, ROS scavenging enzyme genes, and stress-related genes under stress conditions, 2-week-old hydroponically grown rice seedlings or Arabidopsis seedlings were treated with 250 mM mannitol for 12 h, non-treated materials were used as the control. All these harvested leaf samples were then rapidly frozen in liquid nitrogen and stored at 80°C for further expression analysis.

Total RNA was extracted from plant samples using the Trizol reagent (TaKaRa, Japan). Reverse transcription was performed using a Prime Script RT reagent kit (TaKaRa). qRT-PCR was performed with a CFX Connect Thermal Cycling System (Bio-Rad, USA) using a SYBR Green PCR Master Mix kit (TaKaRa). The gene expression levels were normalized with the reference gene *OsActin1* in rice and the reference gene *AtActin2* in Arabidopsis. The fold-change in the expression of each target gene was calculated by using the 2^−ΔΔCt^ method. Primer information for the qRT-PCR assay is included in Supplementary Table 1.

### Statistical Analysis

All data in this work were obtained from at least three biological replicates with three technical replicates each. Statistically significant differences were determined by one-way ANOVA, followed by Tukey's test (*P* < 0.05).

## Accession Codes

The gene sequence data in this study can be found in the Rice Annotation Project Database (RAP-DB, http://rapdb.dna.affrc.go.jp/) and Arabidopsis Database (TAIR, https://www.arabidopsis.org) under the following accession numbers:

UGT85E1 (Os02g0755500), OsABA1 (Os01g0737800), OsABA2 (Os03g0810800), OsABA4 (Os01g0128300), OsNCED1 (Os02g0704000), OsbZIP23 (Os02g0766700), OsABI5 (Os01g0859300), OsRAB16 (Os01g0702500), OsRAB21 (Os11g0454300), OsDREB2B (Os02g0656600), OsDREB1A (Os09g0522200), OsAPX2 (Os07g0694700), OsCATA (Os02g0115700), OsCATB (Os06g0727200), OsFeSODb (Os06g0143000), OsActin1 (Os03g0718100), AtRD29A (AT5G52310), AtRD29B (AT5G52300), AtDREB1A (AT4G25480), AtDREB1B (AT4G25490), AtActin2 (AT3G18780).

## Data Availability Statement

The original contributions presented in the study are included in the article/Supplementary Material, further inquiries can be directed to the corresponding author/s.

## Author Contributions

B-kH and QL conceived the experiments and wrote the paper with inputs from all co-authors. QL, G-rD, Y-qM, and S-mZ performed the research. QL, XL, X-kL, and Y-jL analyzed the data. All authors contributed to the article and approved the submitted version.

## Funding

This research was supported by grants from the National Natural Science Foundation of China (No. 31970290 to B-kH).

## Conflict of Interest

The authors declare that the research was conducted in the absence of any commercial or financial relationships that could be construed as a potential conflict of interest.

## Publisher's Note

All claims expressed in this article are solely those of the authors and do not necessarily represent those of their affiliated organizations, or those of the publisher, the editors and the reviewers. Any product that may be evaluated in this article, or claim that may be made by its manufacturer, is not guaranteed or endorsed by the publisher.
