# Supplementary material for: Rice Glycosyltransferase Gene UGT85E1 Is Involved in Drought Stress Tolerance Through Enhancing Abscisic Acid Response
Source: Front Plant Sci. 2021 Dec 23;12:790195. doi: 10.3389/fpls.2021.790195 (PMC8733621; doi:10.3389/fpls.2021.790195)
Supplement: Supplementary file 1 [file Data_Sheet_1.docx]

**Supplemental Figure**

**Supplemental Figure 1**. **Generation and identification of *UGT85E1* mutant lines.** *ugt85e1* mutants (ko-8, ko-9) were generated by the CRISPR/Cas9 system. A: ko-8 is a mutant with one guanine deletion in *UGT85E1* coding region; B: ko-9 is another mutant with two guanines deletion in *UGT85E1* coding region.

Table S1. Primers used in this study

| For gene cloning | |
| --- | --- |
| C-UGT85E1- F | GGTACCATGGGCTCAAACTCGCGCCTGC |
| C-UGT85E1- R | GGATCCTCAGCTTGCGTGATCACCTCTT |
| UGT85E1-cas9-F | GTCACGTCACGCAGGACATCCCCA |
| UGT85E1-cas9-R | AATGGGGATGTCCTGCGTGACGTGC |
| For mutant identification | |
| UGT85E1-detect-F  UGT85E1-detect-R | tgggctcaaactcgcgcctg  CAGGCGCGAGTTTGAGCCCA |
| For real-time PCR analysis of different genes | |
| OsActin1-F | GACCTTGCTGGGCGTGAT |
| OsActin1-R | GTCATAGTCCAGGGCGATGT |
| UGT85E1 -F | ATGGGCTCAAACTCGCGCCT |
| UGT85E1 -R | CAGGCGCGAGTTTGAGCCCA |
| OsNCED1-F  OsNCED1-R | ACCATGAAGTCCATGAGGCT  TCTCGTAGTCTTGGTCTTGG |
| OsABA1-F  OsABA1-R | GGATGCCATTGAGTTTGGTT  TGGCTGACTGAAGTCTCTCG |
| OsABA2-F  OsABA2-R | GTGCAAGCAGCTCGAGCTTGAGC  AGCGCAACCTTGCTTTCCAACGG |
| OsABA4-F  OsABA4-R | CATCCTCTACGCCTACCTG  GAACATCCTGACGATCCCC |
| OsDREB2B-F  OsDREB2B-R | GCACCTCTCCTGCTCCTCC  GCTGCTGCTCTCCTCCTCG |
| OsABI5-F  OsABI5-R | CATTTTCCTTGCCGCTACCG  CAATCATCATCCCATTCCCA |
| OsRAB16-F | CACACCACAGCAAGAGCTAAGTG |
| OsRAB16-R | TGGTGCTCCATCCTGCTTAAG |
| OsRAB21 -F | CACACCACAGCAAGAGCTAAGTG |
| OsRAB21-R | TGGTGCTCCATCCTGCTTAAG |
| OsbZIP23 -F | GGAGCTGAACGATGAACTCCAG |
| OsbZIP23 -R | TCGGCTCATTCTCTCTAGAACCTC |
| OsDREB1A- F | ATGTATGGTCCCACAGCACG |
| OsDREB1A- R | ACAACACAGCTGGCCCATTA |
| OsCATA -F | TTGGGGGTGAAGATTGCGAA |
| OsCATA -R | CGACAACAGAAGATGCGTGC |
| OsCATB-F | GCTTGCACAGTTTGACAGGG |
| OsCATB-R | CGACTGTGGAGAACCGAACA |
| OsAPX2-F | TTCAGCTTTCGTTTGTGCGG |
| OsAPX2-R | ACCACTCGCAATCCAACGAT |
| OsFeSODb-F | ACAACGGCAACCCATTACCA |
| OsFeSODb-R | CAGCCAGACCCCAAAAGTGA |
| AtRD29A-F  AtRD29A-R | ATACCATTCGGAGCAGGA  ATACCATTCGGAGCAGGA |
| AtRD29B-F  AtRD29B-R | ATACCATTCGGAGCAGGA  TGGCAGTCATCAGTGTGA |
| AtDREB1A-F  AtDREB1A-R | CTTCACAACAGCTAATCGAG |
|  | CGCACTCCATTTCAGTACCA |
| AtDREB1B-F  AtDREB1B-R | TTGGGCTGGACAAGGAGAGGATGA  AGGACGACGGTCTCAACGGTGA |
| AtActin2-F  AtActin2-R | TCCCTCAGCACATTCCAGCA  GATCCCATTCATAAAACCCCAGC |
